# Venous thromboembolism prophylaxis in postacute care units: a health record review

**DOI:** 10.1016/j.rpth.2026.103349

**Published:** 2026-01-13

**Authors:** Pil Joo, Kathleen Qu, Jacob Stasso, Tenaaz Irani, Sanmeet Chahal, Tim Ramsay, Lana Castellucci

**Affiliations:** 1 Department of Family Medicine, University of Ottawa, Ottawa, Ontario, Canada; 2 Ottawa Hospital Research Institute, Ottawa, Ontario, Canada; 3 Faculty of Medicine, University of Ottawa, Ottawa, Ontario, Canada; 4 Department of Internal Medicine, University of Ottawa, Ottawa, Ontario, Canada

**Keywords:** anticoagulants, post-acute care, venous thromboembolism prophylaxis

## Abstract

**Background:**

Venous thromboembolism (VTE), encompassing deep vein thrombosis (DVT) and pulmonary embolism (PE), is a major cause of vascular-related mortality. While VTE prophylaxis (VTEp) is well established in acute care settings, its value in postacute care units, such as transitional care units (TCUs) at our institution, remains unclear.

**Objectives:**

This study therefore aimed to characterize current VTEp use in TCUs at a tertiary care hospital and to evaluate associated thrombotic and hemorrhagic outcomes.

**Methods:**

We conducted a retrospective electronic health record review of patients admitted to TCUs at The Ottawa Hospital between March 1, 2021, and March 1, 2022. Patients on therapeutic anticoagulation for indications other than VTEp or with repeat TCU admissions were excluded. Data were collected with standardized forms and analyzed descriptively; logistic regression was used to explore associations between VTEp and thrombotic or hemorrhagic complications.

**Results:**

Among 1218 patients (mean age, 76 years; 56% female), 72% received VTEp during their TCU stay—primarily enoxaparin (85%) or unfractionated heparin (12%); 75% of these patients remained on prophylaxis for their entire TCU admission. VTEp recipients more often had recent surgery or active cancer, whereas patients in the non-VTEp group more commonly had dementia or previous gastrointestinal bleeding. The overall PE incidence and DVT incidence within 90 days were low (0.8% and 0.9%, respectively) and similar between the groups. Hemorrhagic complications, intracranial hemorrhage (0.4%), and gastrointestinal bleeding (0.9%) were likewise infrequent and comparable. Adjusted odds ratios for these outcomes included 1.0 with wide CIs.

**Conclusion:**

VTEp was used frequently and was usually continued for the full TCU stay. The number of VTE and bleeding events were low, limiting the ability to determine the optimal risk-benefit balance of VTEp use in this population.

## Essentials

• Little is known about the practice patterns and effectiveness of anticoagulants used to prevent venous thromboembolism (eg, pulmonary embolism and deep vein thrombosis) during the immediate postacute care period, when patients remain in the hospital after their acute medical needs have been addressed.
• We conducted a health record review at a tertiary care hospital in Canada.
• We found that the majority of patients in this population received venous thromboembolism prophylaxis, often continued for the duration of their stay in postacute care units.
• Thrombotic and bleeding events were rare; as a result, we could not determine whether anticoagulant use in this context reduces or increases these outcomes.

## 1 Introduction

Venous thromboembolism (VTE), which includes deep vein thrombosis (DVT) and pulmonary embolism (PE), is the third-leading cause of vascular-related mortality worldwide [1]. The annual VTE incidence in Canada is estimated at 1 to 2 per 1000 persons, approximately 40,000 to 80,000 new cases each year [[2], [3], [4]]. Similar incidence rates were reported in the United States and France [5,6]. For acutely or critically ill inpatients, VTE prophylaxis (VTEp) has clearly reduced mortality and morbidity [7]. However, evidence to guide VTEp use in the immediate postacute care hospital populations, such as patients in transitional care units (TCUs) of The Ottawa Hospital (TOH), is limited. Patients whose acute care needs are met, but cannot be discharged home, are typically transferred to the TCUs while waiting for further discharge planning. This knowledge gap is important because TCU patients are typically older, frailer, and less mobile, all of which are factors that increase both VTE risk and bleeding risk, which complicates the decision regarding the use of VTEp in this population [[8], [9], [10]].

Our literature review revealed that most clinical practice guidelines for VTEp are derived from acute care data, raising doubts about their applicability to TCUs and other postacute care settings. Several risk-stratification models for VTE are available; however, these were developed for acute care settings, and their applicability to postacute care populations remains uncertain [[11], [12], [13]]. Moreover, previous studies have reported inconsistent VTEp prescribing patterns in these populations [14]. Notably, there is still little direct evidence on the effectiveness of VTEp for postacute care patients [15,16].

When deciding whether to prescribe VTEp, physicians must balance thromboembolic risk against potential adverse events, particularly bleeding [17,18]. Given the inconsistent practice and absence of tailored guidance, TCU patients may face preventable harm. To address this knowledge gap, we carried out a health record review to examine VTEp use in our institution’s TCUs.

## 2 Methods

### 2.1 Study design, setting, and population

We conducted a retrospective, electronic health record review of all patients admitted to the TCUs at TOH, a 1100-bed tertiary academic hospital located in Ottawa, Canada. The setting of this study was TOH’s 3 TCUs comprising approximately 200 beds. Patients who cannot be discharged home safely from medical and surgical acute care inpatient units are transferred to TCUs while waiting for further disposition planning. In our TCUs, medication orders continue automatically from the originating acute care units unless TCU physicians intentionally discontinue them. Adults transferred to a TCU between March 1, 2021, and March 1, 2022, were considered. Patients receiving anticoagulants for other reasons than VTEp were excluded, such as long-term anticoagulant therapy for atrial fibrillation and new diagnosis of VTE in the acute care unit prior to TCU transfer. Patients who were transferred to TCU multiple times during the study period were included only once, in their first transfer. Institutional approval was obtained from the Ottawa Health Science Network Research Ethics Board (OHSN-REB).

### 2.2 Protocol

Because this was a health record review with a primary objective to describe current VTEp practices, no formal sample size calculation was performed. We reviewed the records of all patients meeting the inclusion and exclusion criteria within a 1-year period. We requested a formal search for medical records from the TOH Data Warehouse fulfilling the inclusion criteria. We then applied exclusion criteria to identify cases to be included in the final analysis. We followed robust methodological standards for chart reviews, including abstractor training, definition of variables a priori, use of a standardized case record form, development of a codebook, and evaluation of interrater reliability [19]. A standardized data abstraction form was developed on the RedCap platform and further refined via pilot data from January and February 2021 [20].

Patient demographics, including age, sex, past medical diagnoses, as well as recent medical events during the acute care unit stay, were captured using the search function of the electronic health record system with relevant, predefined keywords. In addition, all discharge notes from the acute care units, nursing flow sheets, and medication administration records were manually reviewed.

### 2.3 Outcomes

The primary outcome was to characterize current VTEp practices in the TCUs, such as the proportion of patients who experienced VTEp during the TCU stay and the duration of and agent used for VTEp therapy. The secondary outcomes included the proportions of patients who developed symptomatic PE, DVT, intracranial hemorrhage, gastrointestinal hemorrhage, and all-cause death and their odds ratios in the groups with and without VTEp.

To capture these events, we used the electronic health record system at TOH and reviewed charts for up to 90 days following transfer from the TCU, including outpatient records during the postdischarge period. For the diagnoses of PE or DVT, we required confirmatory evidence from computed tomography, ultrasound, or thrombosis service consultation notes indicating a new diagnosis. Intracranial hemorrhage was recorded only when supported by computed tomography imaging, and gastrointestinal hemorrhage required endoscopic confirmation. TOH is the designated referral center for VTE under an established protocol and is the only center with neurosurgical service in the extended region, helping ensure capture of relevant outcome events. There were no surveillance programs for VTEs or hemorrhagic complications for TCU patients during this period, and thus, we assume the positive diagnoses reflect symptomatic presentations.

For VTEp use, we reviewed the medication administration record to identify administration of heparin, enoxaparin, dalteparin, tinzaparin, fondaparinux, rivaroxaban, apixaban, dabigatran, or edoxaban. Patients were categorized as receiving VTEp if any of these anticoagulants were administered for prophylactic purposes at any point during their TCU stay. To distinguish prophylactic from therapeutic anticoagulant use, we searched for a set list of diagnoses that would require therapeutic dosing, such as atrial fibrillation, PE, and DVT. When cases were unclear, classification was resolved by consensus at regular meetings.

### 2.4 Statistical analysis

Descriptive statistics are reported. Categorical variables are summarized as frequencies (%), and continuous variables are presented as the means ± SDs or with medians and IQRs for nonnormally distributed data. Logistic regression was used to report adjusted odds ratios comparing thrombotic and hemorrhagic outcomes between the VTEp group and the non-VTEp group. For thrombotic complications, the logistic regression models included age, sex, recent surgery, active cancer, previous VTE, and recent cerebrovascular accident as covariates. For hemorrhagic complications, the models included age, sex, a history of previous hemorrhagic complications, hemoglobin levels, and platelet levels. For the logistic regression, we excluded patients with a code status of 4 (comfort care only), as these patients are often palliative and frequently do not receive prophylactic treatment. For interrater agreement, 51 randomly chosen cases were reviewed by duplicate abstractors. There were 6 pairs of abstractors among the 4 abstractors, and each abstractor assessed 24 to 26 randomly chosen cases of the 51 cases. Cohen κ calculation was attempted. However, there was often perfect agreement between 2 raters (which results in 0/0 and thus not a number). For this reason, we report the percentage agreement between 2 pairs of observations. Because this was an exploratory audit of practice, hypothesis testing was not performed; therefore, no *P* values are presented. All analyses were carried out in R (version 4.2.0; R Core Team) [21].

## 3 Results

### 3.1 Patient characteristics

A total of 1218 patients met the inclusion criteria (Figure). The mean age was 76 ± 13 years, and 56% were females. Overall, 65% of transfers originated from medical services and 32% from surgical services; 28% of the cohort had undergone surgery during the index acute care stay or within the preceding 12 weeks. Additional baseline characteristics are presented in Table 1. Duplicate abstraction of 51 charts yielded ≥87% agreement across key variables, including 97% concordance for the VTEp administered variable (Supplementary Table 1).

**Figure:**
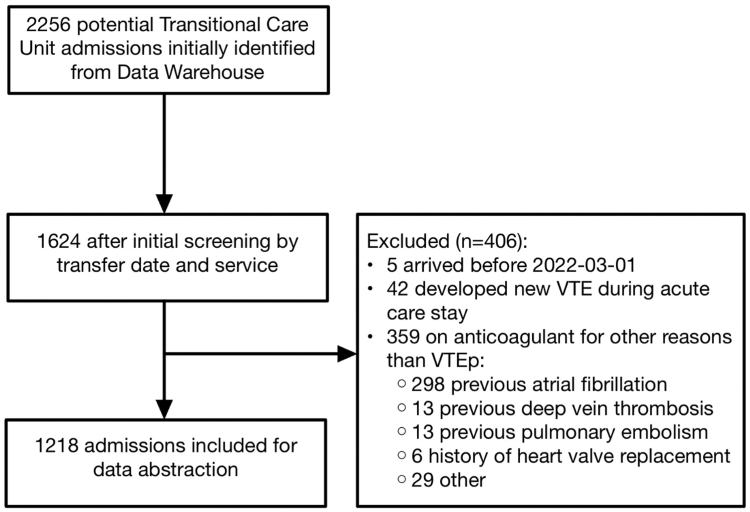
Study flow diagram. VTE, venous thromboembolism; VTEp, venous thromboembolism prophylaxis.

**Table 1:** Demographics.

Characteristic	All (*N* = 1218)	Given VTEp (*n* = 874)	Given VTEp excluding code status 4 (*n* = 833)	No VTEp (*n* = 344)	No VTEp excluding code status 4 (*n* = 267)
Age at transfer to TCU (y), mean (SD)	76.1 (13.3)	75.5 (13.6)	75.2 (13.7)	77.7 (12.2)	76.5 (12.6)
Female	56 (676)	56 (487)	56 (468)	55 (189)	54 (144)
Residence prior to admission					
Home	79	81	82	75	78
Group home	2.90	2.20	2.20	4.70	5.20
Retirement home	14	14	13	15	12
Long-term care	2.40	1.70	1.40	4.10	3.00
Other	1.40	1.10	1.20	2.00	1.90
Code status at arrival					
1 (full code)	50	55	57	36	46
2 (DNR but ICU)	10	11	12	7.60	9.70
3 (DNR, no ICU)	33	32	31	36	44
4 (comfort care only)	6.50	1.10	NA	20	NA
Unclear	<0.1	0.10	0	0	0
Code status at discharge					
1 (full code)	47	53	55	34	44
2 (DNR but ICU)	10	11	12	7.00	9.00
3 (DNR, no ICU)	33	32	33	37	47
4 (comfort care only)	9.40	4.50	NA	22	NA
Unclear	<0.1	0.10	0	0	0
Originating acute service					
Medical	65	59	59	81	79
Surgical	32	39	40	13	14
Intensive care	0.70	0.80	0.70	0.30	0.40
Other	2.00	0.70	0.50	5	6.30
TCU length of stay (d), median (IQR)	14 (7-29)	13 (7-27)	13 (7-26)	17 (8-38)	20 (9-41)
Surgery during acute care admission or within 12 wk prior to TCU transfer	28	35	35	11	13
Mobility at the time of transfer					
Independent	28	24	25	40	49
1-person assist	34	37	37	26	27
2-person assist	34	36	35	29	22
Bed rest	3.00	2.40	2.40	4.70	0.80
Unknown/unspecified	0.60	0.70	0.70	0.30	0.40
Mobility at the time of discharge					
Independent	38	35	36	47	58
1-person assist	27	30	30	19	20
2-person assist	26	26	26	25	18
Bed rest	8.80	8.40	7.10	9.60	3.40
Unknown/unspecified	0.70	0.70	0.70	0.60	0.70
Dementia	30	24	23	46	46
Diabetes	31	31	30	32	32
Congestive heart failure	10	10	9.50	12	12
Hx of myocardial infarction	8.90	9.30	9.50	7.80	7.50
Chronic kidney disease	17	18	18	17	15
Active cancer	13	14	13	10	6.40
Antiplatelet use (on transfer to TCU)	37	39	39	31	35
Previous VTE					
PE	0.90	1.10	1.20	0.30	0
DVT	1.70	1.70	1.80	1.70	1.90
PE and DVT	0.40	0.60	0.60	0	0
VTE not specified	0.20	0.30	0.40	0	0
None	97	96	96	98	98
Hx of cerebrovascular accident	25	23	23	28	25
Hx of intracranial hemorrhage	8.30	7.80	7.80	9.60	6.70
Hx of gastrointestinal bleeding	5.70	4.50	4.60	8.70	8.60

Values are % or % (*n*) unless specified.

DNR, do-not-resuscitate; Hx, history; ICU, intensive care unit; NA, not available; TCU, transitional care unit; VTEp, venous thromboembolism prophylaxis.

Among the 1218 patients, 874 (72%) received VTEp during their TCU stay. The demographic parameters were largely similar between the groups; however, the VTEp group had more recent surgical transfers and higher rates of active cancer, whereas the non-VTEp group had more code status level 3 (full medical management and do-not-resuscitate, no intensive care unit transfer) designations and a greater history of gastrointestinal bleeding and dementia.

The median TCU length of stay was 14 days (IQR, 7-29 days). Patients who did not experience VTEp stayed longer (median, 17 days) than those who did (median, 13 days). The VTEp cohort also contained a greater proportion of individuals requiring 1- or 2-person assistance for mobility, whereas patients in the non-VTEp group were more often independent at transfer (Table 1).

### 3.2 Prophylactic regimens

Most prophylaxed patients received a single anticoagulant, predominantly enoxaparin (85%) or unfractionated heparin (12%) (Table 2). Twelve patients (1.4%) required a second agent during their stay. Among these 12 patients, 4 involved a second anticoagulant order overlapping in time with the first anticoagulant, and 5 patients involved agents being changed but without a time lapse in between.

**Table 2:** Anticoagulants used (% of 874 patients given anticoagulants).

Name	Anticoagulant 1, % (*n*)	Anticoagulant 2^a^, % (*n*)
Enoxaparin	85 (745)	0.5 (4)
Heparin	12 (108)	0.6 (5)
Rivaroxaban	1.0 (9)	0.2 (2)
Tinzaparin	0.7 (6)	
Apixaban	0.3 (3)	
Fondaparinux	0.2 (2)	
Dalteparin	0.1 (1)	0.1 (1)

a Second anticoagulants were used among 12 patients.

Among patients who received prophylaxis, 75% (654/874) remained on VTEp for their entire TCU admission. When examining those who received VTEp for at least 90% of their TCU days, this proportion increased to 82% (720/874). Conversely, only 2.4% (21 patients) received VTEp for <10% of their TCU days.

### 3.3 Thrombotic and hemorrhagic outcomes

Table 3 provides a comparison of thrombotic and hemorrhagic complications and deaths in the first 90 days after TCU transfer. PE occurred in 0.8% (10/1 218) of the cohort, and DVT occurred in 0.9% (11/1218) of the cohort; the incidence rates were similar between the VTEp and non-VTEp groups. Intracranial hemorrhage and gastrointestinal bleeding were infrequent (0.4% and 0.9%, respectively) and were not different between the groups. These thrombotic and hemorrhagic complications were comparable regardless of whether VTEp was administered.

**Table 3:** Complications within 90 days from TCU transfer.

Complications	All (*N* = 1218), % (*n*)	Given VTEp excluding code status 4 (*n* = 833), % (*n*)	No VTEp excluding code status 4 (*n* = 267), % (*n*)
Pulmonary embolism	0.8 (10)	0.7 (6)	1.1 (3)
Deep vein thrombosis	0.9 (11)	1.0 (8)	1.1 (3)
Intracranial hemorrhage	0.4 (5)	0.5 (4)	0 (0)
GI hemorrhage	0.9 (11)	0.8 (7)	0.7 (2)
Death	10 (125)	4.8 (40)	2.6 (7)

GI, gastrointestinal; TCU, transitional care unit; VTEp, venous thromboembolism prophylaxis.

Half of the PEs (5/10) and just over half of the DVTs (6/11) were diagnosed within 30 days of transfer. When PE or DVT were combined, 17 (1.4% of the total) patients experienced a VTE event within 90 days of transfer. Among these 17 patients, the median time to PE diagnosis was 30 days from admission (IQR, 24.25-56 days), whereas the median time to DVT diagnosis was 28 days (IQR, 26-51.5 days). Adjusted logistic regression analyses revealed no statistically significant associations between VTEp use and thrombotic or hemorrhagic outcomes; CIs for all odds ratios crossed 1.0 (Table 4, Supplementary Table 2).

**Table 4:** Odds ratios of 90 days complications when given VTEp.

Complications	Unadjusted odds ratio	Adjusted odds ratio^a^
Pulmonary embolism	0.64 (0.17-3.04)	0.43 (0.10-2.19)
Deep vein thrombosis	0.85 (0.24-3.92)	0.59 (0.15-2.84)
Intracranial hemorrhage	Not available^b^	Not available^b^
GI hemorrhage	1.12 (0.27-7.57)	1.40 (0.32-9.83)
Death	1.87 (0.88-4.62)	1.56 (0.70-4.00)

GI, gastrointestinal; VTEp, venous thromboembolism prophylaxis.

a Logistic regression model included the following as covariates—pulmonary embolism/deep vein thrombosis: age, sex, recent surgery, active cancer, previous venous thromboembolism, and recent cerebrovascular accident; intracranial/GI hemorrhage: age, sex, previous hemorrhagic complications, hemoglobin, and platelet; and death: age, sex, recent surgery, active cancer, previous venous thromboembolism, preadmission residence, do-not-resuscitate, antiplatelet, recent cerebrovascular accident, previous hemorrhagic complications, and recent falls.

b No intracranial hemorrhage observed in the nonprophylaxis group.

## 4 Discussion

A majority (72%) of the 1218 TCU patients received VTEp, and three-quarters (75%) of those individuals remained on prophylaxis for their entire stay. This finding suggests that VTEp is commonly used in our TCUs and, if prescribed at the time of acute care unit to TCU transfer, it is typically continued until discharge.

The 2 most common prophylactic agents were enoxaparin and unfractionated heparin. Consistent with guideline-directed prescriptions, VTEp recipients had more classical risk factors, such as recent surgery, active cancer, and impaired mobility, whereas patients with non-VTEp more often had contraindications such as gastrointestinal bleeding or dementia. The non-VTEp subgroup may represent patients transferred to the TCU primarily for social reasons, such as the need for placement in a long-term care facility. Conversely, the VTEp group may consist more of postoperative patients with better preadmission functional status.

The tendency to continue prophylaxis beyond the acute care phase likely reflects clinicians’ uncertainty in the absence of postacute care–specific guidance. Our adjusted models did not show any obvious association with VTEp and VTE events or hemorrhagic complications, due to low event rates, resulting in wide CIs.

Several risk-stratification models such as the Padua, modified IMPROVE (International Medical Prevention Registry on Venous Thromboembolism), and Geneva scores [[11], [12], [13]], classify acutely ill medical inpatients as high risk when the predicted 90-day VTE probability exceeds approximately 1% to 3%. In our cohort, the 90-day VTE incidence was 1.4%, despite 72% prophylaxis coverage, a figure that falls at or below these thresholds. A 2024 multicenter analysis revealed that these risk prediction models poorly discriminated between high- and low-risk groups, with an adjusted 90-day VTE incidence of 2.1% irrespective of risk category or prophylaxis use [22]. These data question the applicability of hospital-based tools to postacute care settings.

The only large trial extending prophylaxis into the postdischarge period—a 2013 study comparing 10 ± 4 days of enoxaparin with 35 ± 4 days of rivaroxaban—demonstrated a modest reduction in composite VTE (relative risk, 0.77; *P* = .02) at the cost of enrolling >8000 participants and including asymptomatic events [23]. Whether such benefit translates to contemporary TCU populations remains uncertain.

Guidelines mirror the paucity of evidence. The 2012 American College of Chest Physicians guidelines recommend against routine prophylaxis in chronically immobilized nursing home residents (grade 2C) [24], and the 2018 American Society of Hematology update likewise advises no extension of VTEp beyond acute hospitalization [7]. Thrombosis Canada echoes this stance, stating that prophylaxis should generally continue until acute care discharge and not be extended [25]. None of these statements draw on data from dedicated postacute care studies.

We have carefully characterized the use of VTEp in our institution’s postacute care units. To our knowledge, this is the first such description in the literature, particularly within the Canadian context. However, this study was conducted at a single institution, and the findings may not be generalizable to other postacute care settings. In addition, our ability to interpret any association between VTEp use and thrombotic or hemorrhagic complications is limited by the observational design, low event rates, and multiple potential confounders (such as confounding by indication), as well as the absence of prespecified hypothesis testing. It is also possible that some thrombotic and hemorrhagic events were not captured if patients moved away or presented to another hospital, and established regional referral pathways were not followed. Prospective, multicenter studies are needed to develop a calibrated risk model for postacute care patients and to test whether selective prophylaxis improves outcomes without increasing harm.

In summary, there is currently no validated method for predicting VTE risk in postacute care patients. Our study demonstrated a low number of VTE and bleeding events at 90 days, limiting the ability to determine the optimal risk-benefit balance of VTEp use in this population. In the absence of clinical guidelines recommending extended use of VTEp in the postacute care patient population, a careful balance of individual VTE and bleeding risk factors need to be considered. Prospective, multicenter studies are needed to develop a calibrated risk model for postacute care patients and to test whether selective prophylaxis improves outcomes without increasing harm.

## 5 Conclusion

VTEp is frequently used in our TCUs and typically continues for the duration of admission. The limited number of VTE and bleeding events in those receiving VTEp and those not using VTEp highlights the need for robust, postacute care–specific guidelines. Use of routine VTEp in the postacute care context should be weighed against the lack of postacute care unit–specific guidelines, the absence of validated risk-stratification tools, and the absence of any known efficacy estimate for VTEp in this setting. Our single-center audit provides a foundation for multicenter prospective studies to validate these observations.
