# Supplementary material for: Venous thromboembolism prophylaxis in postacute care units: a health record review
Source: Res Pract Thromb Haemost. 2026 Jan 13;10(1):103349. doi: 10.1016/j.rpth.2026.103349 (PMC12964025; doi:10.1016/j.rpth.2026.103349)
Supplement: Supplementary Tables 1 and 2 [file mmc1.docx]

# **Supplementary Table 1 Interrater agreement**

| **Question** | **Number of paired observations** | **Number of observations with same value** | **Agreement** |
| --- | --- | --- | --- |
| Arrival date* | 54 | 47 | 87% |
| Date of birth | 54 | 50 | 93% |
| Sex | 36 | 35 | 97% |
| Given VTEp? | 36 | 35 | 97% |
| VTEp = Venous thromboembolism prophylaxis  *6 out of 7 discrepancies were of 1 day difference | | | |

**Supplementary Table 2 Odds ratios of 90 days complications when given VTEp with initial models**

|  | **Unadjusted odds ratio** | **Adjusted odds ratio^&^** |
| --- | --- | --- |
| Pulmonary embolism | 0.64 [0.17 – 3.04] | 0.33 [0.02 – 9.58] |
| Deep vein thrombosis | 0.85 [0.24 – 3.92] | 0.31 [0.04 – 2.65] |
| Intracranial hemorrhage | Not available* | Not available* |
| GI hemorrhage | 1.12 [0.27 – 7.57] | 0.28 [0.03 – 3.01] |
| Death | 1.87 [0.88 – 4.62] | 0.79 [0.25 – 3.02] |
| VTEp = venous thromboembolism prophylaxis, GI = gastrointestinal  *No intracranial hemorrhage observed in non-prophylaxis group.  ^&^Logistic regression model included as covariates: *age, sex, recent surgery, active cancer, previous venous thromboembolism, pre-admission residence, do-not-resuscitate, bedrest at transfer, bmi, antiplatelet, recent cerebrovascular accident, previous hemorrhagic complications, recent falls* | | |
